# Single-photon quantum router with multiple output ports

**DOI:** 10.1038/srep04820

**Published:** 2014-04-28

**Authors:** Wei-Bin Yan, Heng Fan

**Affiliations:** 1 Beijing National Laboratory for Condensed Matter Physics, Institute of Physics, Chinese Academy of Sciences, Beijing 100190, China

## Abstract

The routing capability is a requisite in quantum network. Although the quantum routing of signals has been investigated in various systems both in theory and experiment, the general form of quantum routing with many output terminals still needs to be explored. Here we propose a scheme to achieve the multi-channel quantum routing of the single photons in a waveguide-emitter system. The channels are composed by the waveguides and are connected by intermediate two-level emitters. By adjusting the intermediate emitters, the output channels of the input single photons can be controlled. This is demonstrated in the cases of one output channel, two output channels and the generic N output channels. The results show that the multi-channel quantum routing of single photons can be well achieved in the proposed system. This offers a scheme for the experimental realization of general quantum routing of single photons.

Quantum routing^1^,^2^,^3^,^4^,^5^,^6^,^7^,^8^,^9^ of information from one sender to many receivers is an essential function for the quantum network^10^. Single photons are suitable candidates as the carrier of quantum information due to the fact that they propagate fast and interact rarely with the environment. The field-matter interaction can be used to manipulate the photons. By the way, the light-light interaction by the platform of linear optics^8^,^9^,^11^,^12^ can also be used to manipulate the photons. The photon transport in a one dimensional (1D) waveguide has been studied extensively both in theory^13^,^14^,^15^,^16^,^17^,^18^,^19^,^20^,^21^,^22^,^23^,^24^,^25^,^26^ and in experiments^27^,^28^,^29^,^30^,^31^,^32^,^33^,^34^,^35^,^36^ because the strong coupling of the waveguide-emitter can be achieved. In the waveguide-emitter system, the waveguides act as the channels and the emitters as the nods of the quantum network. Based on these advantages, the quantum routing of photons in the waveguide-emitter system is promising. Recently, two output channel quantum routing of single photons is studied theoretically in a waveguide-emitter system^6^,^7^. The input single photons can be redirected into either of the two output channels with a maximal probability of unity and no more than 1/2, respectively. It is interesting if the photon can be redirected into either of the output channels with an extremely high probability. Moreover, a general form of many output channel quantum routing of the single photons in the waveguide QED system will be of considerable interest.

For these purposes, we propose a novel scheme for the quantum routing of single photons from one input waveguide into N output waveguides. In our scheme, the ith output waveguide is connected with the input waveguide by an intermediate two-level system (TLS). After scattering, the single photon injected into the input channel is redirected into other channels. For the single output channel quantum routing, the quantum interferences redirect the input photon into the output channel completely when the intermediate TLS resonantly interacts with the input and output channels with the same strength. The single output channel routing properties can be modified when an additional TLS is coupled to the input channel. In the two output channel case, the photon can be redirected into either of the output channels with an approximate unity probability. The photon can also be redirected completely into both the output channels with various probabilities. In the generic N output channel case, the quantum interferences prevent the photon being redirected into the other channels except the input channel when all the TLSs resonantly interact with the channels with equal strengths for a large value of N. They also can completely prevent the photon being directed back into the input channel for suitable parameters for any value of N. The photon can be redirected into a desired channel with an approximate unity probability.

The schematic diagram of the system under consideration is shown in Fig. 1. The input channel is a Sagnac interferometer^37^,^38^,^39^ composed by a semi-infinite waveguide, a 50:50 beam splitter and a waveguide loop. The input channel connects the *i*th infinite waveguide by an intermediate TLS at the middle point of the waveguide loop. For simplicity, we label the interaction position *x* = 0. It is convenient to bring in the even and odd operators as 
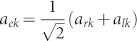
 and 
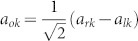
, with *a_rk_* (*a_lk_*) being the annihilation operator for the right (left)-moving mode with the frequency *v_g_*|*k*| in the waveguide. Hereafter we will take the photon group velocity *v_g_* = 1. The clockwise- and counterclockwise-moving photons in the waveguide loop can be treated as the left- and right-moving photons. If we inject a photon into the input waveguide, an even mode quasi particle can be produced at the middle point of the waveguide loop when the phases of the clockwise- and counter clockwise-moving photons are equal. In the even and odd picture, the odd mode only contributes to the free energy part in the Hamiltonian. Therefore, it is enough to study the dynamics of the even mode. For simplicity, we omit the superscript *e* of the even operator *a_ek_*. The even part of the Hamiltonian can be written as (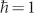) 
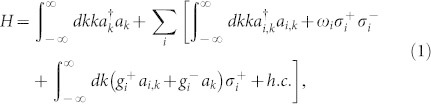
with 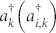 being the even mode creation operator in the input (*i*th output) waveguide, and *ω_i_* being the *i*th TLS transition frequency. We have taken the energies of the TLS ground states zero. The terms of the second line in Hamiltonian (1) represent the interaction of the TLSs with the waveguides. 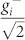 and 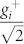 are coupling strengths of the *i*th TLS to the input waveguide and the *i*th output waveguide, respectively. The coupling strengths can be written as 
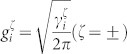
, with 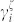 being the decay rate from the *i*th TLS to the waveguides. The TLS can be a real two-level atom or an artificial two-level system. In the single-photon case, the TLS can also be a cavity or a cavity-atom dressed system. The transition frequency of the TLS can be tuned by Stark shift and the coupling strength between the waveguide and the TLS can be varied by changing the distance between them^40^,^41^. Especially, for a waveguide-atom dressed system, the effective coupling strength of the dressed TLS to the waveguide can be adjusted by the extra laser^42^. We have made two approximations: one is to extend the frequency integration to ±∞, the other is to assume that the coupling strengths are independent of the frequencies, which is equivalent to the Markovian approximation. These approximations are valid since we will focus on the pulse with a narrow frequency width around the carrier frequency.

The arbitrary state of the system in the single-excitation subspace has the form of 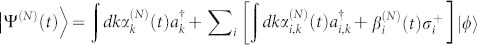, with 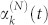, 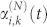 and 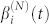 being the probability amplitudes. The state |*ϕ*〉 represents that all the waveguides and TLSs contain no excitation. We assume that, initially, a photon prepared in a wave packet with a Lorenzian spectrum is injected into the input waveguide, while the TLSs and the output waveguides contain no excitation, i.e. 
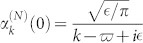
, 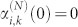, 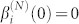. Here 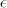 and *ϖ* are the spectral width and the center frequency of the input wave packet, respectively. When the width 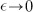, the frequency of the carrier is equal to the central frequency. This corresponds to the monochromatic limit.

## Results

### Single output channel

**Figure 2:**
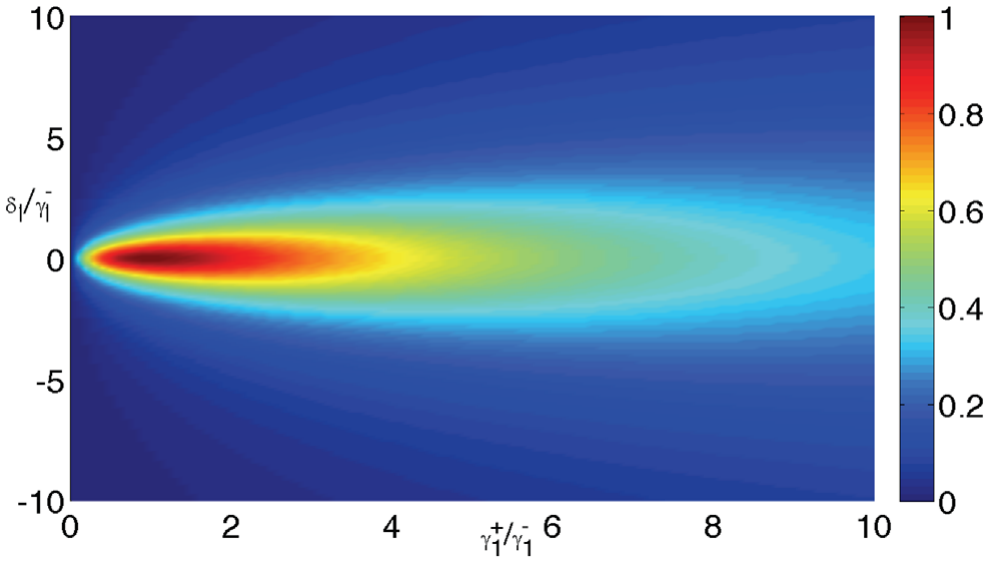
Probability of the single photon in the 1st output channel in the long-time limit 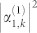 against the detuning and decay rate when *N* = 1.

When the input channel is connected with only one output channel by a TLS, the probability amplitudes are 
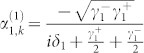
, and 
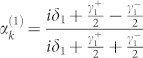
, with *δ_i_* = *ω_i_* − *k*. Obviously, only when 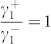 and *δ*_1_ = 0, the input photon is redirected into the output channel completely. This is due to the quantum interferences. When the detuning *δ*_1_ is large enough, the photon will be back into the input channel with an approximate unity probability. Here we bring in a parameter 
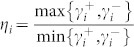
 to measure the difference between the coupling strengths of the *i*th TLS to the input and the *i*th waveguides. When 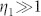, we can get 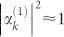, which is similar to the large detuning case. Although the decay rate of the TLS to the input channel is much smaller than the decay rate to the output channel, the photon is prevented being redirected into the output channel. The condition *η*_1_ → ∞ corresponds to the limit that one of the two waveguides is decoupled to the TLS. In this case, the input and output channels are not connected, and the photon will be back into the input channel completely. To show the details of the quantum routing of the single phonon in the *N* = 1 case, we plot the probability 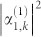 against the detuning and decay rates in Fig. 2. The probability 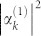 is not plotted here because 
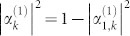
. The probabilities of the photon in the input and output channels can be controlled by adjusting the detuning and coupling strengths. The single photon can be directed into either of the two channels completely or directed into the two channels with a desired probability. The *N* = 1 case is analogous to a waveguide coupled to a Λ-type three-level quantum emitter^25^,^43^,^44^.

### Two output channels

**Figure 3:**
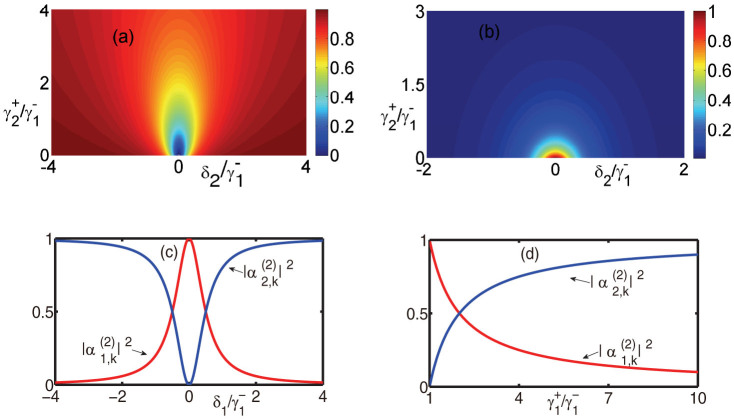
Probabilities 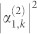, 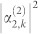 and 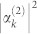 against the detunings and coupling strengthes when *N* = 2. (a) and (b) are the probabilities 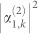 and 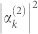 when *δ*_1_ = 0 and 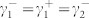, respectively. (c) and (d) are the probabilities 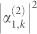 and 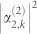. (c) is the probabilities against the detuning *δ*_1_ when 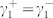, 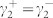, and 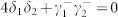. (d) is the probabilities against the coupling constant 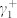 when *δ*_1_ = *δ*_2_ = 0, and 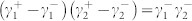.

When the input channel is connected with two output channels by two TLSs, the probability amplitudes are obtained as 
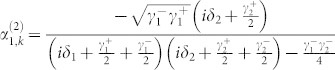
, and 
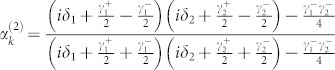
. Because the expression of 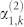 is similar to 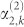, the study of the properties 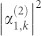 and 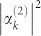 is enough. The large detuning between the 1st TLS and the input photon prevents the photon being redirected into the 1st channel. Especially, when *δ*_2_ = 0 and 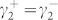, it also prevents the photon being directed into the input channel. As a result, for an extremely large value of *δ*_1_, the photon will be redirected into the 2nd channel with an approximate unity probability when *δ*_2_ = 0 and 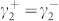. And when both the detunings *δ*_1_ and *δ*_2_ are large enough, the photon will be directed into the input channel with an approximate unity probability. Besides, the photon distribution can be influenced significantly by the coupling strengths. For example, when *δ*_2_ = 0 and 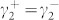, the large value of 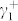 prevents the photon being directed into both the input and 1st channels. When *η*_1_ = *η*_2_ = 1 and *δ*_1_ = *δ*_2_ = 0, the input photon will be directed into the input channel with a small probability and be redirected into the other two channels averagely with a large probability. To see the details, we plot the probabilities 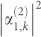 and 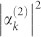 against the detuning and coupling strength of the 2nd TLS when *δ*_1_ = 0 and 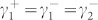 in Fig. 3(a) and 3(b). It is interesting that when *δ*_2_ and 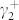 is small enough, the photon will be back into the input channel with an extremely high probability. This will be studied in detail below.

If we do not wish the input photon to be back into the input channel, it is easy to choose the appropriate parameters which satisfy 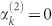. Here we take two simple cases to investigate it. One case is when 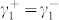 and 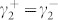, the condition 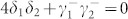 can be satisfied for various values of the detunings. The other case is when *δ*_1_ = *δ*_2_ = 0, the relation 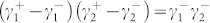 can be easily satisfied. Especially, in the latter case, when 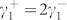 and 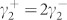, the input single photon is redirected into the two output channels with equal probability 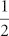. We plot the probabilities of the photon being redirected into each of the two output channels in the two cases in Fig. 3(c) and 3(d). It shows that the input photon can be completely redirected into the output channels with various probabilities.

It is necessary to study a special case of *N* = 2, that is, the 2nd TLS is decoupled to the 2nd output channel, i.e. 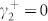. This can be understood that an additional TLS is coupled to the input channel in the *N* = 1 case. The additional TLS will modify the system behavior. For example, when *η*_1_ = 1 and *δ*_1_ = 0, we find 
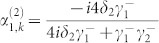
 and 
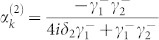
. Hence, when the additional TLS resonantly interacts with the input photon, i.e. *δ*_2_ = 0, the photon is directed into the input channel compared with the *N* = 1 case, in which the photon is redirected into the 1st output channel. This can be seen in Fig. 3(a) and 3(b). When *δ*_2_ is large enough, the input photon will be almost completely redirected, mapped to the *N* = 1 case. By adjusting the additional TLS, the photon distribution in the input and output channels can be controlled. This provides more control to the single output channel case.

### *N* output channels

We proceed to study the general case that the input channel connects with *N* output channels by *N* TLSs. Let's first consider the simplest case that all of the *N* output channels are identical. That is to say, all the TLS are identical, all the decay rates to the input channel are identical, and all the decay rates to the output channels are identical. We label *δ_i_* = *δ*, 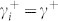, and 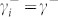. The probability amplitudes are obtained as 
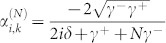
, and 
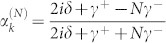
. It is noted that when all the TLSs interact resonantly with the input photon and the coupling strengths satisfy *γ*^+^ = *Nγ*^−^, the interferences prevent the input photon being directed into the input channel and redirect the input photon into each of the *N* output channels with equal probability 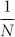. When all the coupling strengths are equal and all the detunings are zero, the probabilities are obtained as 
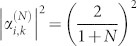
 and 
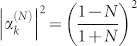
. As discussed above, the quantum interferences redirect the input photon completely from the input channel into the other channel when *N* = 1. As the number of the output channels increases, the probability of the photon back into the input channel increases. When *N* = 3, the input photon is distributed in each of the four channels, including the input and output channels, with equal probability 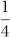. When the number of the output channels is large enough, the quantum interferences direct the input photon back into the input channel almost completely.

We now study a simple case to illustrate how to redirect the input photon into the desired channel. We assume that *N* − 1 of the *N* channels are identical except the *m*th channel. we label *δ_i_*_≠*m*_ = *δ*′, 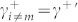, and 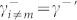. The probability amplitudes are obtained as 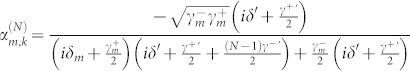 and 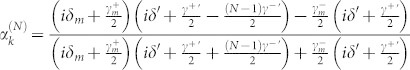. When the decay rates 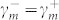 are much larger than other parameters, 
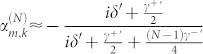
. The photon will be redirected into the *m*th channel almost completely when 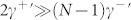. The limit of this condition is that the *N* − 1 TLSs are decoupled to the input channel. Besides, when all the detunings are large enough, the photon will be directed into the input channel with an approximate unity probability. When 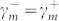, *δ_m_* = 0, and *δ*′ is large enough, the photon will be redirected into the *m*th channel with an approximate unity probability. Similar to the *N* = 2 case, the photon can be redirected completely into the output channels where the photon probabilities can be controlled. As a consequence, the *N* output channel quantum routing of the single photons can be achieved.

The single photons are considered as the ideal carrier of quantum information. The quantum information can be encoded by the routed photons. We label two degenerate photon modes as *a*_1_ and *a*_2_. For example, the two degenerate modes can be horizontally and vertically polarized modes or can be right and left circularly polarized modes. To achieve the quantum routing of the quantum information, we bring in *N* extra intermediate TLSs to make sure that each output port is connected with the input port by two intermediate TLSs. The distance between the two intermediate TLSs is much smaller than the wavelength of the photon. One of the two intermediate TLSs only interacts with *a*_1_-mode photon and the other only interacts with *a*_2_-mode photon. In this case, the Hamiltonian can be written as 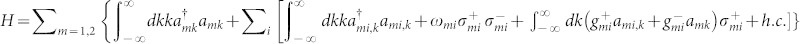. To study the quantum routing of the quantum information, we take the input state as 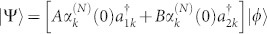, with |*A*|^2^ + |*B*|^2^ = 1. Obviously, when 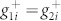, 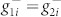 and *ω*_1*i*_ = *ω*_2*i*_, the input state |Ψ〉 can be routed to the desired channel with desired probability. In this case, the information remains unchanged after being routed. In our scheme, the control information is stored in the coupling strength and the TLSs. It will be interesting to use quantum information to control the routing. This might be achieved by preparing connections between the control quantum states and the target quantum states.

The authors in Ref. 9 have raised a set of five requirements imposed on a full quantum router. It will be interesting when a router meets all the requirements. As discussed above, Our scheme will meet all those requirements if the control information can also be encoded into quantum states. This full quantum routing scheme in various systems is an interesting question which need be further studied.

## Discussion

We have investigated the quantum routing of the single photons from one channel to multiple output channels in the proposed system. We have shown that the input photon can be redirected into any of the channels with a high efficiency in our scheme. Alternatively, by cascaded combining many two output channel routers demonstrated in the presented literatures, the multiple output channel quantum routing can be achieved. We note that our general *N* output channel quantum routing shows essential advantages compared with the cascaded combined one. In the cascaded combined scheme, the efficiency of the *i* output channel quantum routing is limited by the efficiency of the *i* − 1 output channel quantum routing. However, our scheme is immune to these drawbacks. It is necessary to point out that our scheme is equivalent to that the input waveguide loop is coupled to *N* output waveguide by an intermediate *N* + 1-level system at the middle point of the waveguide loop. Our scheme sheds light on the experimental realization of quantum routing.

## Methods

The continuum form of Hamiltonian (1) can be derived from its discrete form by following the technique in Ref. 45, 46. The free Hamiltonian of the photon in the 1D cavity of length *L* has the form of 
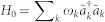
, and the interaction Hamiltonian of the photon with the TLS has the form of 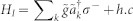. The sum can be converted into an integral by 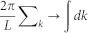. As done in Ref. 45, we can bring in the operator 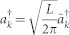 and obtain the Hamiltonian (1) with continuum form.

The Shrödinger equation governed by Hamiltonian (1) gives a set of differential equations of probability amplitudes. By performing the Laplace transformation, the set of differential equations under the initial condition are transformed as 
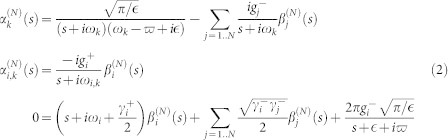
We proceed to obtain the expression of 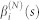. For simplicity, we label 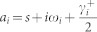, 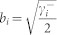, and 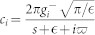. The last equation in equations (2) can be written as 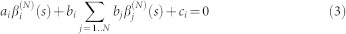
From equation (3), we can find 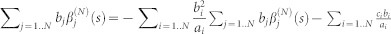 to obtain the expression of 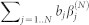. Consequently, the expression of 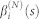 can be obtained as 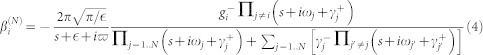
From the equation (4) and equations (2), the expressions of 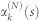 and 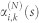 can be easily found.

In the long-time limit, the amplitudes can be found by performing the inverse Laplace transformation of 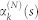, 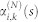 and 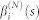. Then we prove that the amplitude 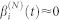 when *t* → ∞. It means that the TLSs are in their ground states in the long-time limit due to the interaction with the continuum of modes. Obviously, 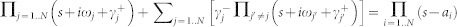, with *a_i_* being the root of the equation 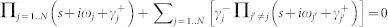. We lable *a_i_* = *x_i_* + *iy_i_*, with *x_i_* and *y_i_* being real numbers. We will show that the value of *x_i_* can only be smaller than zero.

If *x_i_* ≥ 0, we can get 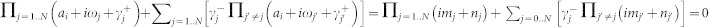, with *m_j_* and *n_j_* being real numbers and *n_j_* > 0. We label 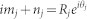 and Π*_j_*_ = 1.*N*_(*im_j_* + *n_j_*) = *Re^iθ^*, and get 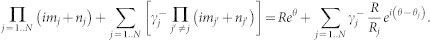
Obviously, it can not be zero because 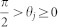 or 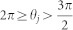, and 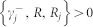. Therefore, the value of *x_i_* must be smaller than 0. As a consequence, we can find 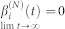.

Similarly, the probability amplitudes of the photon in each channel in the long-time limit are found as 
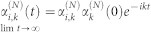
 and 
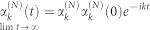
, with 
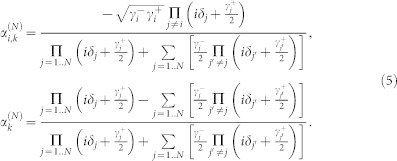
We have taken the conversation of energy condition *ω_i_*_,*k*_ = *ω_k_*. After scattering, the probabilities of the single photon in each channel are 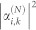 and 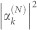.

## Author Contributions

W.-B.Y. and H.F. proposed the model, calculates and analyzed the results. W.-B.Y. and H.F. co-wrote the paper.

## Figures and Tables

**Figure 1:**
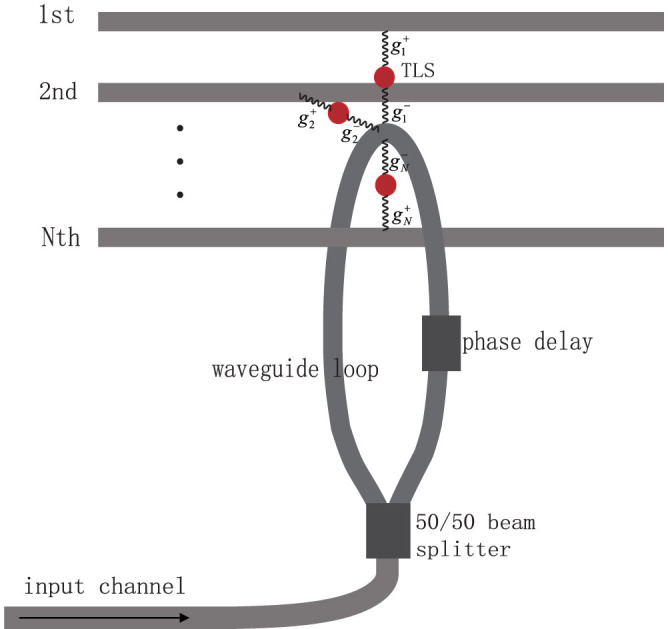
Schematic diagram of the multi-channel quantum routing of the single photons. *N* 1D waveguides are connected with the input channel by *N* intermediate TLSs.
